# Methods in Microbial Biodiscovery

**DOI:** 10.3390/md19090503

**Published:** 2021-09-03

**Authors:** Angela A. Salim, Zeinab G. Khalil, Ahmed H. Elbanna, Taizong Wu, Robert J. Capon

**Affiliations:** Institute for Molecular Bioscience, The University of Queensland, St. Lucia, QLD 4072, Australia; a.salim@uq.edu.au (A.A.S.); z.khalil@uq.edu.au (Z.G.K.); ahmed.elbanna@pharma.cu.edu.eg (A.H.E.); taizong.wu@uq.edu.au (T.W.)

**Keywords:** microbial biodiscovery, microbial isolation, media MATRIX, chemical profiling, molecular networking, artifacts, microbial natural products

## Abstract

This review presents an account of the microbial biodiscovery methodology developed and applied in our laboratory at The University of Queensland, Institute for Molecular Bioscience, with examples drawn from our experiences studying natural products produced by Australian marine-derived (and terrestrial) fungi and bacteria.

## 1. Introduction

For over a billion years, the global microbiome has evolved to encompass seemingly limitless genetic and taxonomic diversity. Complex microbial communities featuring a bewildering array of species of bacteria and fungi survive and prosper in ecosystems, ranging from deep ocean sediments to near shore reefs; beach sands to estuarine muds; fertile savannah soils to barren desert landscapes; and rainforests to deep canyons to alpine mountains. Indeed, microbes can be found living on or in every animal, insect and plant species on the planet, not to mention every sediment, sand, mud, soil and rock. Microbes are the ultimate survivors and colonisers.

In addition to species diversity, natural selection relentlessly drives adaptations in microbial metabolism, with successive generations acquiring ever more diverse defensive natural products (i.e., antibiotics), capable of enhancing survival in complex and highly competitive communities. Over millennia, the global microbiome has come to host a vast arsenal of ecologically and biologically potent natural products, encoded within a myriad of biosynthetic gene clusters (BGCs).

For the latter half of last century, science and industry successfully exploited these microbial chemical defences, fuelling a knowledge revolution that inspired new pharmaceuticals and agrochemicals, driving global commerce and improving quality of life. Microbe-inspired products included some of the most widely recognised and successful pharmaceuticals, spanning antibiotics (e.g., penicillins, tetracyclines, streptomycins, vancomycins and rifampicin), antilipidemics (e.g., statins), immunosuppressives (e.g., cyclosporins, rapamune and tacrolimus) and anticancer agents (e.g., adriamycin). They also included some of the most effective agrochemicals in history, including antifungals (e.g., strobilurins), antiparasitics (e.g., avermectins), insecticides (e.g., spinosins) and herbicides (e.g., glufosinate). The journey to detect, isolate, identify, evaluate, develop, manufacture and market microbe-inspired products heralded a golden age in global science, business, healthcare and agriculture.

Notwithstanding this success, when confronted by lower returns on investment and the perception of a near-exhausted microbial resource, late-last-century industry turned elsewhere for inspiration. Two decades on, and confronted by surging levels of drug resistance and community demand for safer and more effective medicines and agrochemicals, the need for inspiration remains as urgent as ever.

Reinvigorated by scientific and technical advances, there is a strong case that microbes can once again play a prominent role. Such optimism arises on multiple fronts, including genomic discoveries documenting a near limitless reservoir of transcriptionally inactive (i.e., silent) BCGs, dominant in virtually all microbial genomes, as well as the prevalence of microbial diversity in marine ecosystems. Overlooked by science and industry last century, these resources have the potential to fuel a microbe-inspired renaissance in drug discovery. To deliver on this bonanza requires robust and effective methods for tapping in to these hidden and underexplored genetic/molecular resources. What follows is an account of some of the lessons learned in our laboratory at The University of Queensland, Institute for Molecular Bioscience.

## 2. Microbial Diversity

### 2.1. Substrate Diversity

As noted above, microbes colonise virtually all substrates, providing a limitless and bewildering choice for natural products researchers. A few selected examples of Australian terrestrial and marine substrates/microbes that, in our hands, have yielded exciting new chemistry in are listed below.

#### 2.1.1. Examples of Terrestrial Substrates and Microbes

**Figure 1:**
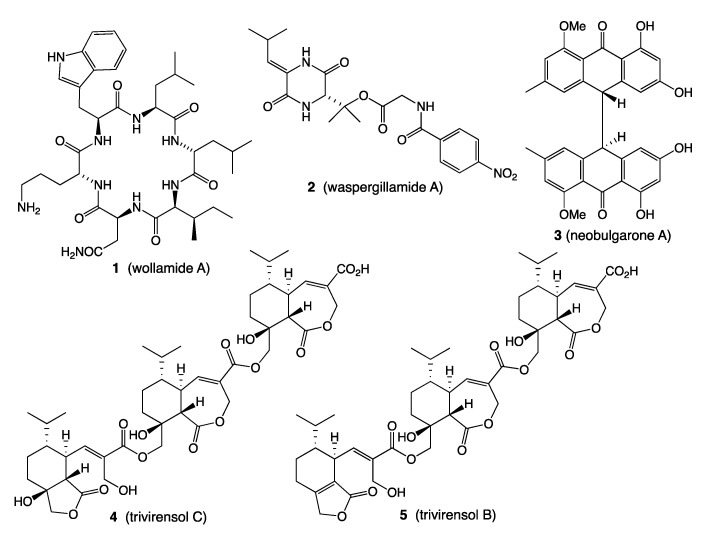
Terrestrial natural products **1**–**5**.

*A. Soil: Streptomyces* nov. sp. MST-115088 isolated from an arid soil sample collected near Wollogorang Station in the gulf country of Northern Queensland returned a new class of cyclic hexapeptide, such as wollamide A (**1**) (Figure 1), currently under development as an antibiotic against multidrug resistant (MDR) tuberculosis [1,2].

*B. Wasp: Aspergillus* sp. CMB-W031 isolated from a mud dauber wasp collected from an urban location in Brisbane, Queensland, returned waspergillamide A (**2**) (Figure 1), the first reported example of a natural depsi-tetrapeptide diketopiperazine, and the first natural diketopiperazine to incorporate either a 3-hydroxy-valine or a *p*-nitrobenzamide residue [3].

*C. Wasp nest: Penicillium* sp. CMB-MD22 isolated from a mud dauber wasp nest collected from an urban location in Brisbane, Queensland, returned a family of new *syn* and *anti* bianthrones, such as neobulgarone A (**3**) (Figure 1), with highly selective antifungal activity against a co-isolated fungus, suggestive of a natural anti-infective chemical defence for wasp larvae [4].

*D. Termite nest: Trichoderma virens* CMB-TN16 isolated from a termite nest collected from an urban location in Brisbane, Queensland, returned a selection of new sesquiterpene dimers and trimers examples of which were natural pro-drugs, including trivirensol C (**4**) undergoing in situ transformation to trivirensol B (**5**), exhibiting bacteriostatic properties against vancomycin resistant Enterococci (VRE) (Figure 1) [5,6].

#### 2.1.2. Examples of Marine Substrates and Microbes

**Figure 2:**
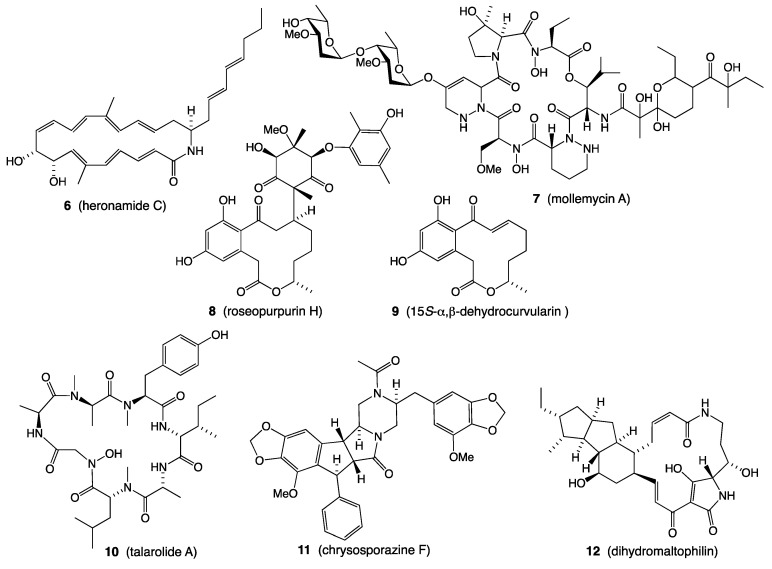
Marine natural products **6**–**12**.

*A. Beach sand: Streptomyces* sp. CMB-M0406 isolated from shallow water beach sand (−1 m) collected off Heron Island, Queensland, returned an unprecedented family of polyketide macrolactams, inclusive of heronamide C (**6**) (Figure 2), which elicited a dramatic and reversible non-cytotoxic effect on mammalian cell morphology [7,8].

*B. Deeper water sediments: Streptomyces* sp. CMB-M0244 isolated from sediment (−55 m) collected off South Molle Island, Queensland, returned a first in class glyco-hexadepsipeptide-polyketide, mollemycin A (**7**) (Figure 2), which exhibited exceptionally potent growth inhibitory activity against Gram-positive and Gram-negative bacteria (IC_50_ 10–50 nM) and drug-sensitive (3D7, IC_50_ 7 nM) and drug-resistant malaria (Dd2, IC_50_ 9 nM) [9].

*C. Mangrove sediment: Penicillium roseopurpureum* CMB-MF038 isolated from a mangrove mud sample collected in the Boondall Wetland Reserve, Queensland, returned a selection of new polyketides, with roseopurpurin H (**8**) being a rare example of a natural Michael adduct prodrug, undergoing in situ intracellular reverse Michael addition to yield the cytotoxic Michael acceptor 15*S*-α,β-dehydrocurvularin (**9**) (Figure 2) [10].

*D. Tunicate: Talaromyces* sp. CMB-TU011 isolated from a marine tunicate collected from intertidal waters off Tweed Head, New South Wales, returned a new cyclic heptapeptide, talarolide A (**10**) (Figure 2), bearing an exceptionally rare hydroxamate moiety [11].

*E. Fish: Chrysosporium* sp. CMB-F294 isolated from the gastrointestinal tract of a Brisbane, Queensland, market-purchased fish, returned an new class of phenyl propanoid piperazine, including chrysosporazine F (**11**), exhibiting inhibitory activity against the multidrug efflux pump P-Glycoprotein with a potency > 2-fold that of the positive control verapamil (Figure 2) [12,13].

*F. Cone snails:* A selection of *Streptomyces* spp. isolated from the stomach and hepatopancreas of five Conus species collected from Lady Musgrave Island on the Great Barrier Reef, Queensland, all yielded the same blend of antifungal polycyclic tetramic acids, including dihydromaltophilin (**12**) (Figure 2), consistent with highly conserved inter-kingdom evolution and chemical ecology [14].

In addition to the Australian substrates listed above, we have had success examining substrates from other countries, ranging from deep Venezuelan caves to Chilean Antarctic territory soils, as well as sediments from the deep below the South China Sea (−2500 m). The take-home lesson from our experience is that, while substrates obtained at great expense (and risk) from remote and adventurous locations can certainly deliver, so can more readily accessible substrates, even those found close to home.

### 2.2. Microbial Isolation

The microbial diversity associated with any plant, animal, insect or inorganic substrate typically far exceeds that which can be readily cultured in the laboratory, and which can subsequently be recovered and cryopreserved as pure isolates. Those microbes that are overlooked are often (mis)categorised as “unculturable”, which implies that they defy any effort at lab cultivation. Perhaps a more apt characterisation would be that a significant portion of microbial diversity remain “uncultured” under given lab culture conditions, but that this might change under different lab conditions. Variables that can impact on whether a given microbe is culturable or unculturable include the method of processing and applying the substrate to a solid-phase isolation plate, the composition of the isolation plate culture media, the temperature and duration of cultivation and the presence or absence of fast-growing competing microbes. The challenge facing natural product researchers is to select a practical range of substrate processing and culture conditions, to best optimise the chance of recovering pure isolates representative of maximum microbial diversity, while operating within the finite constraints of available time, space and resources. Some useful considerations and methods include the following:

#### 2.2.1. Substrate Processing

*Heat Shock:* A portion of substrate (1 g) suspended in sterile water (20 mL) is heated for 20 min at 55 °C in a water bath. Then it is vigorously shaken and the supernatant serially diluted 10-, 100- and 1000-fold. Aliquots (50 μL) of each dilution are applied with an inoculation loop to separate agar isolation plates (i.e., Petri dishes), which are sealed with parafilm, labelled and incubated at 30 °C.

*Stamping Method:* A portion of dried substrate (~1 g) in a sterile mortar is ground lightly with a pestle, and the residue is pressed into a sterile foam plug (14 mm in diameter). The agar isolation plate is inoculated by stamping the foam plug eight or nine times in a circular fashion, giving a serial dilution effect. After sealing with parafilm and labelling, agar isolation plates are incubated at 30 °C.

*Homogenization Method:* In the case of substrates consisting of small portions of animal, plant or insect tissues, it is preferable to use a sterile mortar and pestle to homogenise in sterile water (~5 mL). Then, after centrifuging, apply the serially diluted supernatant to agar isolation plates, using either the heat shock or stamping methods described above.

*Cutting and/or Streaking Method:* In the case of substrates consisting of larger portions of animals or plant tissues not readily amenable to homogenisation, two alternate options are to (i) carefully streak over the surface of the agar isolation plate and/or (ii) aseptically cut into smaller pieces and place these on an agar isolation plate, and then seal, label and incubate at 30 °C.

*Animal Inner Tissues:* The recovery of microbes from animal inner tissues requires surface washing and sterilization, followed by dissection to access inner tissues, which can be processed and applied to isolation plates using either the homogenization, or the cutting and/or streaking methods described above.

#### 2.2.2. Cultivation Conditions

*Bacteria and/or Fungi:* It is important to decide at the outset whether you are interested in isolating bacteria and/or fungi. If only bacteria, you can add a potent antifungal (e.g., cycloheximide) to kill all fungi, and if only fungi, you can add a potent antibacterial (e.g., ampicillin) to kill all bacteria. In our lab, we elected to isolate both bacteria and fungi. To achieve this, we add sub-minimum inhibitory concentrations (MIC) of antifungals and antibacterials to isolation plates, to slow down the growth of faster growing microbial “weeds”, to allow time and space for slower growing strains to make an appearance.

*Petri versus Multi-Well Plates:* For particularly problematic substrates, rich in taxonomically diverse fast and slow growing microbes, lower substrate dilution and the use of multi-well microtitre plates can provide physical barriers (well edges) that give slow growing microbes a chance to establish before being overgrown.

*Isolation Culture Media:* As it is necessary to physically pick colonies to isolate pure microbial strains, isolation plates need to be solid phase. The literature abounds with many common solid-phase cultivation media. A selection used in our laboratory include; Tryptic Soy Agar (TSA), International *Streptomyces* Project-2 (ISP-2) and Project-4 (ISP-4), Potato Dextrose Agar (PDA), Sabouraud Dextrose Agar (SDA), Peptone Yeast Glucose agar (PYG agar), Yeast Extract with Supplements agar (YES agar), Czapek Agar (CZA). Rice media is also commonly used to isolate fungi; however, it is important to note that not all rice media are the same (see below).

#### 2.2.3. Recovering Pure Isolates

*Culture Time:* Some microbes can be very slow to grow, such that harvesting an isolation plate too early can see them overlooked. Alternatively, some microbes, especially unicellular bacteria act like weeds, and rapidly overgrow isolation plates making colony picking near impossible. One option is to harvest colonies from an isolation plate at 1–3 weeks, take a digital image and return it to the incubator for another 2 to 3 weeks, while inspecting regularly to see if additional colonies emerge, at which time these can be recovered by a second round of colony picking.

*Colony Picking:* The heat shock method (see above) has the advantage of selectively killing many unicellular bacteria which can grow fast and take over isolation plates. Likewise, the use of sub-MIC doses of antibiotics (see above) can be used to control overgrowth. Manual colony picking requires the use of a probe to sample cells from distinct bacterial/fungal colonies and transfer to a viral charged with sterile water. Each vial can then be used to inoculate a fresh agar cultivation plate, by wiping a loop of liquid across the surface in a zigzag pattern, after which plates are sealed with parafilm, labelled and incubated at 30 °C. Close visual inspection of these cultivation plates should confirm if the picking process has delivered a pure isolate. If not, the process can be repeated.

### 2.3. Microbial Preservation

Once pure bacterial and fungal isolates have been recovered (see above), it is important to preserve them in a non-replicating state for long-term archive. This is typically done by cryopreservation, where aliquots of microbe cells are packaged and stored at −80 °C in a manner that ensures viability on retrieval. Two protocols we have found useful are as follows:

*Glycerol Cryovials:* The target microbe is grown to a healthy and visible cell density on a solid medium (i.e., Petri dish), and a loop of cells transferred into a screw-capped cryovial (1.2 mL) preloaded with of sterile 40% aqueous glycerol (400 μL) and sterile culture broth (400 μL) (i.e., Sabouraud Dextrose broth for fungi and ISP-2 broth for bacteria). The vial is capped and the contents are mixed thoroughly by inverting several times, after which they are held at room temperature for 60 min prior to being moved to −80 °C freezer storage. As recovered vials cannot be re-frozen, it is prudent to generate and archive multiple cryovials per isolate.

*Cryobead Cryovials:* The commercial “Microbank” storage system (Pro-Lab Diagnostics, UK) consists of sterile cryovials containing 20 porous, coloured ceramic beads immersed in a cryopreservative (deionised water, beef extract, peptone, sodium chloride and glycerol). The beads are acid-washed and of a porous nature that allows microorganisms to readily adhere to the bead surface. The general protocol for using cryobeads is as follows: (i) under aseptic conditions, transfer a loop of cells to the cryovial (as detailed above for glycerol cryovials); (ii) seal the cryovial and mix thoroughly by inverting several times; (iii) let stand for 30 s; (iii) pipette off excess cryopreservative, leaving inoculated beads as free of liquid as possible; and (iv) seal the cryovial finger tight and store at −80 °C. The frozen cryovial can be removed from the freezer, opened and one or more beads removed for the purpose of recovering a viable culture. The remaining beads should be quickly returned to the freezer. In this way each cryobead cryovial is equivalent to 20 glycerol cryovials.

### 2.4. Microbial Taxonomy

Although most natural products researchers are not specialists in microbial systematics (taxonomy), modern genetic sequencing makes it relatively easy for non-experts to undertake the rapid, cost effective and largely reliable taxonomic analysis of both bacteria and fungi. This process involves DNA extraction, and polymerase chain reaction (PCR) amplification and sequencing of taxonomically important genetic markers, followed by nucleotide BLAST searches in the NBCI genomic database. Associated online tools facilitate the assembly of phylogenetic trees, and taxonomic assignments to genus, and even species.

*DNA Extraction:* Many commercial kits provide a convenient and cost-effective solution for extracting DNA from 5–7-day broth cultures. On occasion DNA extraction can prove troublesome when cell walls are resistant to lysis buffer. A typical solution is to use vigorous physical disruption methods, such as grinding with liquid nitrogen, agitating with glass beads or sonicating, followed by exposure to the lysis buffer.

*Bacterial Genetic Markers:* The most widely sequenced DNA region for identifying bacteria is the 16S rRNA gene, using standard 27F and 1492R primers in Sanger sequencing.

*Fungal Genetic Markers:* The most widely sequenced DNA region for identifying fungi is the 18S rRNA gene, using standard ITS1 and ITS4 (Internal Transcribed Spacers) primers in Sanger sequencing.

*PCR Amplification:* Following extraction of microbial DNA it is necessary to amplify the abundance of the genetic markers using PCR and primers for genetic markers. A typical PCR mixture (50 μL) consists of genomic DNA (2 μL, 20–40 ng), EmeraldAmp GT PCR Master Mix (2X Premix) (25 μL), primer (0.2 μM, each), and H_2_O (up to 50 μL). PCR is performed while using the following conditions: (i) initial denaturation at 95 °C for 2 min, (ii) denaturation over 40 × 20 s cycles at 95 °C, (iii) annealing at 56 °C for 20 s and (iv) extension at 72 °C for 30 s followed by one cycle at 72 °C for 5 min. In the most primitive form, amplicons (pCR products) can be visualised by gel electrophoresis to determine if the reaction succeeded and if the product mass is consistent with predicted results.

*Genetic Sequencing and Phylogenetic Analysis:* Successful PCR products are typically sent to a genomic sequencing service for purification and sequencing. On receipt of the sequence data, a BLAST analysis is performed, using the NCBI database to identify the closest match for queried DNA fragments (i.e., 16S rRNA for bacteria and 18S rRNA for fungi). Our practice is to use >97% BLAST similarity for taxonomic determinations to genus level, with species level determinations limited to 100% BLAST similarity with a designated type strain. The queried sequence and taxonomic determination is uploaded to the NCBI database, along with details of the source microbe, generating an accession number that tags the isolate and can be used for publication and future reference.

## 3. Transcriptional Activation

### 3.1. Silent Chemical Defences

As noted earlier, microbes have evolved over billions of years in complex microbial communities awash with chemical cues from competing microbes. As individual microbes evolve and acquire BGCs coding for natural products that provide a survival advantage against other microbes, to conserve energy many of these molecular defences are coupled with regulatory systems (i.e., receptors, signalling pathways and transcriptional activators) that render them silent until needed. With microbial genomes accumulating larger and more specialised molecular arsenals over time, it is hardly surprising that many chemical defences (even the best?) were silenced and held in reserve. Effectively hidden in the pre-genomic era of microbial biodiscovery last century, these silent microbial molecular defences represent an untapped resource for future drug discovery. While genome mining approaches such as heterologous expression—the excision and transcription of silent BGCs in alternate hosts hold the promise of unleashing this resource, they have yet to deliver on a scale commensurate with its potential. More approaches are needed, especially those that capitalize on the ability of the host microbe to turn on in situ production of its own chemical defences.

In recent years, a number of non-genomic approaches have been trialled for in situ transcriptional activation of silent BGCs, with two promising approaches being co-cultivation and the use of chemical cues. What follows is a brief account of our experiences in using these approaches.

### 3.2. Co-Cultivation

**Figure 3:**
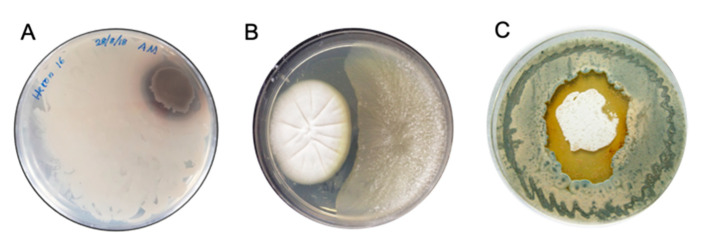
Examples of co-cultivation of (**A**) two bacteria, (**B**) two fungi and (**C**) a bacterium and a fungus: illustrating colour changes at the contact zone, and dominance of one microbe over the other.

Co-cultivation takes advantage of the ability of microbes to sense and respond to the presence (essentially the threat posed by) nearby microbes. The premise behind co-cultivation is that when grown in close proximity, microbe A can detect a chemical cue(s) emanating from microbe B, triggering a transcriptional activation by microbe A of a natural product chemical defensive (i.e., compound A) aimed at repelling microbe B. Importantly, in the absence of a chemical cue(s) from microbe B, the defensive molecule remains transcriptionally silent and inaccessible to natural product researchers. On occasion, this process can also stimulate a counter attack, where microbe B responds to compound A by initiating transcriptional activation of its own chemical defence (i.e., compound B) which it uses in an attempt to repel microbe A. The winner in such a contest is the microbe most capable of mounting an effective chemical ecology inspired chemical defence. When embarking on co-cultivation, some useful considerations include the following:

*(1) Choice of Combatants*: For practical reasons microbial biodiscovery co-cultivations are typically limited to two combatants, bacteria vs. bacteria, fungi vs. fungi, or bacteria vs. fungi (Figure 3). If the two challengers are sourced from different substrates such that it is unlikely they will have encountered each other in the natural world, it is unlikely they will have evolved the ability to detect and respond to each other’s chemical cues and defences. By contrast, our preference is to select co-cultivation combatants from microbes co-isolated from the same substrate—where co-evolution is more likely (although even then not assured).

*(2) Observing Co-Cultivation Events*: Co-cultivation (i.e., chemical ecology) responses are most readily observed when the two challenging microbes encounter each other on a solid-phase (i.e., agar) plate. As the microbial colonies grow together the presence of a chemical response (silent or otherwise) can be evidenced by distinctive colour or morphological changes in one or both microbes in or near to the contact zone, and/or the dominance of one microbe over the other where growth of the latter is suppressed and/or redirected away from the former (Figure 3).

**Figure 4:**
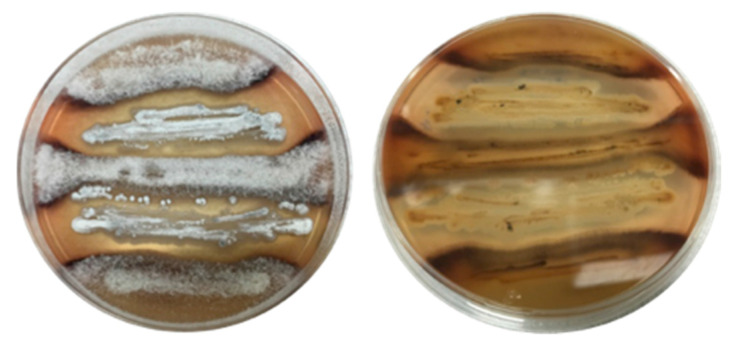
Example of scaled-up solid-phase co-cultivation with a chemical response evident at multiple contact zones. Co-cultivation of fungus CMB-NF041 and bacterium CMB-NB090 (**left** = front side; **right** = back side).

*(3) Scalability*: To access new co-cultivation activated chemical defences it is necessary to scale up the production of biomass in order to produce enough new chemistry to isolate, characterise and solve molecular structures. As the activated chemistry is often only localised within the narrow contact zone (i.e., interface) between microbes, one way to scale up solid-phase production is to inoculate agar plates with each microbe in alternate stripes, generating 2–5 contact zones per plate (Figure 4). Even then it is often necessary to cultivate 50–200 agar plates, which can be very time consuming. An alternate approach is to use broth cultivation; however, this presents other challenges. Different microbes can have different media requirements, leading to wildly different growth rates, to the point where one microbe can dominate a broth co-cultivation, compromising the activation of silent defences. Notwithstanding this possibility, we have encountered many examples where broth co-cultivation can be highly productive. Whether a co-cultivation can make the transition from solid to broth phase cultivation needs to be established on a case by case basis.

**Figure 5:**
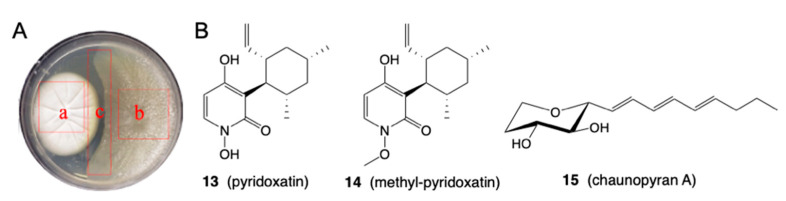
Image of the co-culture agar plate featuring (**A**) CMB-MF028 (a), CMB-MF030 (b) and the growth inhibitory interface (c), along with chemical structures for **13**–**15** (**B**).

The following example of fungus-fungus co-cultivation between *Chaunopycnis* sp. CMB-MF028 and *Trichoderma hamatum* CMB-MF030 illustrates consideration of all the above issues. *Chaunopycnis* sp. CMB-MF028 isolated from the inner tissue of a pulmonate false limpet, *Siphonaria* sp., collected off rock surfaces in the intertidal zone near Shorncliffe, Queensland, returned an array of known and new natural products, including the siderophore pyridoxatin (**13**), known to exhibit antifungal properties [15]. Co-cultivation of CMB-MF028 with the co-isolated fungus *Trichoderma hamatum* CMB-MF030 resulted in a defensive response, where CMB-MF030 transformed **13** to methyl-pyridoxatin (**14**) with a corresponding loss of siderophore and antifungal properties. Countering this, and presumably in response to an as yet unknown chemical cue (i.e., possibly deactivation of its own siderophore **13**), CMB-MF028 activated transcription of a silent BGC coding for chaunopyran A (**15**), the first new example of this rare class of antifungal 2-alkenyl-tetrahydropyrans to be reported in over 30 years (Figure 5). This study illustrates the complexity of offensive and counter-offensive molecular defences encountered during fungal co-cultivation, and the opportunities for accessing new chemistry through biotransformation and transcriptional activation.

### 3.3. Natural Chemical Cues

Building on the phenomena of co-cultivation, yet another approach to transcriptional activation of silent metabolites is to identify natural chemical cues that can be added to monocultures, generating a more easily managed pseudo co-cultivation. The addition of natural chemical cues presents a number of advantages, not the least being the ability to optimize the culture conditions to those of the microbe producing the new chemistry (i.e., media, temperature, duration and soil vs. broth). As such, the use of chemical cues can reduce costs, and increase throughput, reliability and scalability. Three classes of chemical cue that we have developed in recent years are illustrated in the following examples:

*A. Bacterial Lipopolysaccharide (and nitric oxide):* We determined that Gram-negative bacterial cell wall lipopolysaccharide (LPS) could be used as an additive to fungal cultures, to alter the expression of natural products. The LPS effect, whether initiated with commercial samples of LPS or crude autoclaved *E. coli* preparations, could be characterised into four distinct forms:

(i) *Activation*—leading to the production of otherwise silent metabolites.
(ii) *Enhancement*—increasing the levels of production of otherwise minor metabolites.
(iii) *Acceleration*—significantly decreasing the time taken to production of metabolites
(iv) *Suppression*—shutting down the production of metabolites.

**Figure 6:**
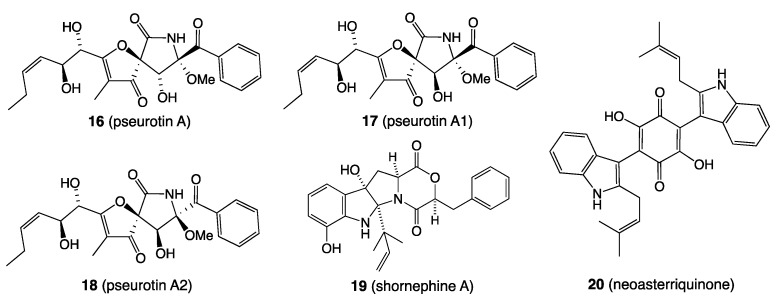
Structures for **16**–**20**.

For example, LPS treatment of *Penicillium* sp. ACM-4616 (sourced from an Australian mushroom) *enhanced* biosynthesis pseurotin A (**16**) and *activated* pseurotins A1–A2 (**17–18**), while LPS treatment of *Aspergillus* sp. CMB-M81F (sourced from a marine sediment collected near Shorncliffe, Queensland) substantially *accelerated* and *enhanced* the biosynthesis of shornephine A (**19**) and *activated* that of neoasterriquinone (**20**) (Figure 6). We employed light and fluorescent microscopy in conjunction with a nitric oxide (NO)-sensitive fluorescent dye and an NO scavenger to provide evidence that LPS stimulation of fungal secondary metabolism coincided with LPS activation of NO. We also demonstrated that 6/40 (15%) of fungi tested were responsive to LPS, and that the effect could be scaled up in broth cultivations [16].

**Figure 7:**
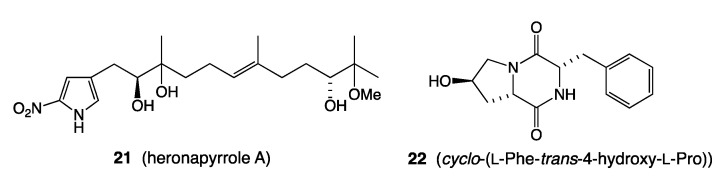
Structures for **21** and **22**.

*B. Diketopiperazines (and nitric oxide): Streptomyces* sp. CMB-M0423 isolated from shallow water beach sand (−1 m) collected off Heron Island, Queensland, returned an unprecedented family of farnesylated 2-nitropyrroles, including heronapyrrole A (**21**) (Figure 7), that exhibited sub-micromolar Gram-positive antibacterial activity [17]. A follow-up study revealed that the CMB-M0423 isolate was contaminated by a co-isolated quiescent fungus, successfully resolved into pure strains of *Streptomyces* sp. CMB-StM0423 and *Aspergillus* sp. CMB-AsM0423. More importantly it was demonstrated that the *Aspergillus* sp. CMB-AsM0423 diketopiperazine *cyclo*-(l-Phe-*trans*-4-hydroxy-l-Pro) (**22**) (Figure 7) acted as a chemical cue to *Streptomyces* sp. CMB-StM0423, stimulating production of nitric oxide that in turn served as a transcriptional regulator for the production of heronapyrroles, which were selectively antifungal against *Aspergillus* sp. CMB-AsM0423. Indeed, in the case of *Streptomyces* sp. CMB-StM0423 the heronapyrroles are silent metabolites, only produced in cultures containing *Aspergillus* sp. CMB-AsM0423 or the diketopiperazine **22**, or an exogenous source of NO [18]. Since making these discoveries we have encountered a bacterial diketopiperazine capable of activating nitric oxide biosynthesis in a fungal pathogen (unpublished), suggestive of a more widespread mechanism for Inter-Kingdom chemical communication.

**Figure 8:**
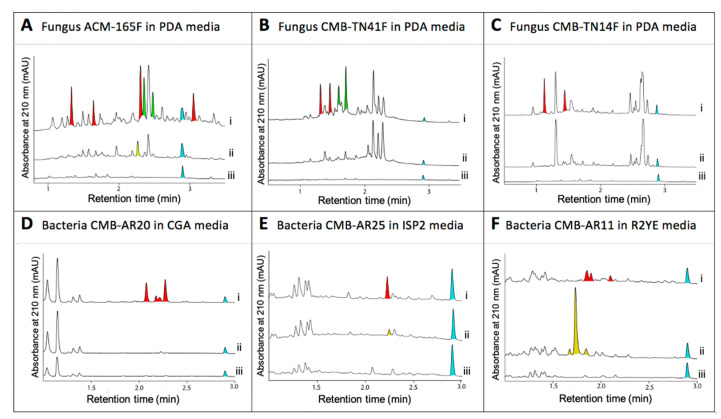
UPLC–DAD (210 nm) traces of (**A**–**C**) fungi and (**D**–**F**) bacteria, where (i) with and (ii) without NOMETA, and (iii) media control (i.e., no inoculation); blue = an internal calibrant; red = activation; green = enhancement; yellow = suppression.

*C. Nitric Oxide Mediated Transcriptional Activation (NOMETA):* Building on the NOMETA concept emerging from Examples 12 and 13, we have carried out additional studies (unpublished), successfully trialling different nitric oxide delivery reagents, to arrive at the optimal reagent, quantity, frequency, timing and method of addition to bacterial vs. fungal cultures. These studies also investigated the influence of variable wavelength LED light panels inside incubators, to enhance in situ (in culture) release of NO, and established a robust workflow capable of detecting a range of NOMETA outputs (Figure 8).

## 4. Biological Profiling

### 4.1. Bioactives

It is generally accepted that, for gene coding for natural products to evolve and persist in the global microbial genome, they should offer the host organism a survival advantage. This could extend to an enhanced ability to compete with and/or defend against other microbes or predatory species (i.e., nematodes); or to sequester metals (i.e., siderophores); or to better enable symbiotic/associative relationships with plants, animals and insects. While ecological properties underpin the pharmaceutical and agrochemical potential of natural products, it is equally true that, for the vast majority of known microbial natural products, very little is known about their real ecological role. Indeed, many microbial natural products reported in the scientific literature are either not attributed any biological property, or, worse, still are described as biologically inactive or are attributed irrelevant biological properties that misdirect from their true potential. Examples of the latter include the viridicatumtoxins, which, although first described and forever badged as mycotoxins, are in fact only weakly cytotoxic, with members of the family being exceptionally potent antibacterials [19]. Given the premise in the opening sentence of this paragraph, we prefer to qualify any claim to a natural product being biologically inactive with a disclaimer specifying “against which biological property(s), as determined by which assay(s), using which experimental method(s) and based on what data”. To discover the true ecological and applied value of microbial natural products, it is essential to make use of biological assays; however, as the range and quality of assays is near limitless, this can be quite challenging. What follows are some thoughts on key bioassay considerations.

### 4.2. Bioassays

#### 4.2.1. Key Bioassays Consideration

When establishing bioassays and reporting on results and conclusions, there are a number of important considerations. Experimental methodology should always be carefully documented, including relevant literature citations; the choice and use of positive and negative controls; and a description of how analytes were prepared and handled, including the choice of solvents, concentrations and volumes, serial dilution ranges, and assay temperature, duration and format (i.e., 96-well plate). It is also critical to run multiple replicates and document how measurements were recorded (i.e., visual inspection for colorimetric or multi-plate readers for cell optical density). If measurements are quantitative (i.e., IC_50_, MIC and LD_99_), then serial dilution curves should be presented with error bars, to both validate data quality and reproducibility. For in vitro assays on extracts and fractions, our preference for quantitative measurements is to use the units μg/mL, whereas for pure compounds (and as chemists) we favour μM. Finally, when interpreting assay results and drawing conclusions, we encourage cautionary use of uncalibrated potency descriptors such as “mild, moderate, significant, remarkable, pronounced, potent etc.”—let the measured value speak for itself, and do not be afraid of concluding that a compound is inactive (albeit with the qualifiers noted above). While there are no hard rules for using potency descriptors such as those listed above, from our perspective, any compound with an in vitro cell cytotoxicity and/or an antibacterial LD_99_ or MIC >30 μM is deemed inactive, 10–30 μM is weakly active, 1–10 μM is noteworthy, <1 μM is significant and <10 nM is potent—albeit with reference to relevant positive controls. While the distinction between such descriptors is largely arbitrary, it is still disturbing to encounter literature reports where IC_50_ is in the high μM even mM range are designated as potent and promising. Likewise, without appropriate documentation of quantitative bioassay methods, and comprehensive characterisation of the purity and structure of analytes, any effort at drawing structural activity relationship (SAR) conclusions must be viewed as questionable.

#### 4.2.2. Outsourcing through Collaboration

It can be costly and time-consuming to establish specialist in lab bioassays, and depending on available space, infrastructure, personnel and expertise many, assay configurations are a step too far for most natural products research groups. This likely explains why natural products researchers are so heavily focused on antibiotic and anticancer bioassays, both of which are simple variants of each other measuring the ability of an extract, fraction or natural product to selectively impede the growth and or kill certain cell types (i.e., carcinoma or pathogen) but not others (i.e., host cells). One approach to upgrade antibiotic assays is to screen against clinical isolates of multidrug-resistant (MDR) pathogens. Likewise, a strategy we have used to upgrade our anticancer assay is to source and screen against a MDR carcinoma cell line overexpressing the drug efflux pump P-glycoprotein (P-gp) the goal being to discover a non-cytotoxic natural product capable of reversing the P-gp mediated MDR phenotype.

An alternative strategy that can enable access to a broader range of indications and diseases, spanning human and animal health, and crop protection targets, is the use of collaborations with academic and industry partners. This is a strategy we have used very effectively over many years to discover natural products with potential value, including in the control of such indications as neurodegenerative (Alzheimer’s and Parkinson’s), infectious (tuberculosis and malaria) and inflammatory (Irritable Bowel Disease) diseases, specific cancer (inhibitors of Sox18 and K-Ras) and pain (isoform selective GlyR potentiators and mu-opioid receptor agonists) targets, as well as endo- and ectoparasites.

### 4.3. Examples of Bioactive Compounds Characterised in Our Lab

**Figure 9:**
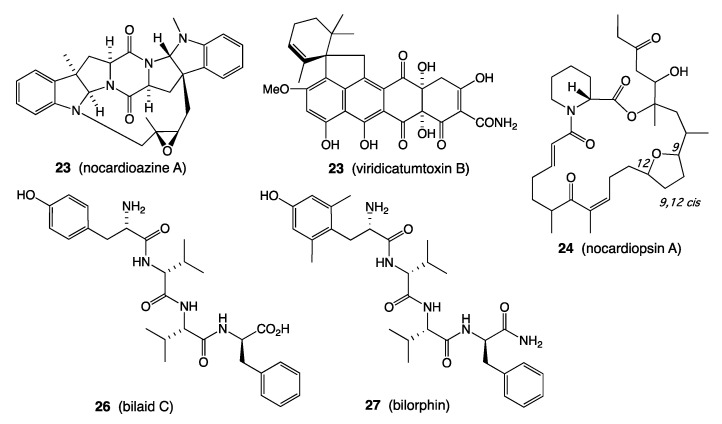
Structures for **23**–**27**.

*A. MDR cancer (P-gp): Nocardiopsis* sp. CMB-M0232 isolated from sediment (−55 m) collected off South Molle Island, Queensland, returned an unprecedented family of isoprenyl bridged diketopiperazines, including nocardioazine A (**23**) which exhibited potent inhibitory activity against the drug efflux pump P-gp (Figure 9) [20].

*B. MDR bacteria: Paecilomyces* sp. CMB-MF010 isolated from inner tissues of an intertidal pulmonated mollusk (*Siphonaria* sp.) collected near Shorncliffe, Queensland, yielded a family of rare and new tetracycline-like polyketides, the viridicatumtoxins, including viridicatumtoxin B (**24**) (Figure 9) that proved to be >270-fold more potent against vancomycin resistant Enterococci (VRE) than the commercial antibiotic oxytetracycline [19]. A subsequent study revealed the viridicatumtoxins to be remarkably more acid stable and resistant to microbial biotransformation than commercial tetracycline antibiotics [21].

*C. Immunosuppressives: Nocardiopsis* sp. CMB-M0232 isolated from sediment (−55 m) collected off South Molle Island, Queensland, returned a family of new macrolide polyketides, such as nocardiopsin A (**25**), as the first new natural examples of the rapamycin class of immunosuppressives to be described in over a decade (Figure 9) [22,23].

*D. Acute pain: Penicillium* sp. MST-MF667 isolated from an estuarine mud sample collected near a boat ramp on the Huon River, Tasmania, returned a diverse range of metabolites including a unique family of tetrapeptide, including bilaid C (**26**) (Figure 9), subsequently developed and patented as the first-in-class analgesic bilorphin (**27**), equipotent to morphine but with an alternate mu-opiod receptor biased signalling [24].

## 5. Chemical Profiling

### 5.1. Dereplication

In microbial biodiscovery dereplication can have different meanings, including (i) the identification of replicate microbial isolates (i.e., identification of duplicates) and (ii) the prioritization of new over known chemistry in complex extracts. As microbial biodiscovery is a numbers game, dereplication needs to be fast, low-cost, robust and effective at tagging microbial replicates, and prioritising new over known, and rare over common natural products. A failure to deploy effective dereplication strategies prior to scale-up cultivations and fractionation risks wasting time and resources in re-isolating and rediscovery known chemistry. Fortunately, significant advances in analytical technologies have led to greatly enhanced dereplication methods drawing on hyphenated technologies which link UPLC and HPLC chromatography to diode array detectors (DAD), as well as various configurations of mass spectrometer including both low and high resolution, inclusive of single-ion extraction (SIE) and tandem mass spectrometry (i.e., QTOF–MS/MS). These technologies can reveal the molecular weight (and even molecular formula) for hundreds of natural products per extract, with MS/MS fragmentations analysed through tools such as Global Natural Products Social (GNPS) molecular networking, revealing structurally related clusters of metabolites [25]. Shown below are case studies where we have used UPLC–DAD or HPLC–DAD–MS, and GNPS molecular networking, to dereplicate and prioritise discovery.

### 5.2. UPLC–DAD and HPLC–DAD–MS

UPLC–DAD or HPLC–DAD–MS can be used to analyse microbial extract generated from pure cultures (isolation plates), to quickly dereplicate duplicate strains, which will have identical chromatograms. A UPLC–DAD based library can also be constructed by extracting the UV–vis spectra from the DAD data, to search for and facilitate the detection of unique chromophores. HPLC–DAD–MS analysis provides the added dimension of mass spectrometry delivering molecular weights and even molecular formula.

### 5.3. Molecular Networking

After the dereplication of duplicate strains, in our hands unique extracts are subjected to UPLC–QTOF to obtain MS/MS data to rapidly dereplicate and prioritise new over known, and rare over common, chemistry. MS/MS data are uploaded to the Global Natural Products Social (GNPS) server developed by researchers at the University of California at San Diego, where it is processed and displayed as a molecular network. GNPS reveals chemical (structural) similarities between natural products within a given sample, or between different samples, based on MS/MS spectral alignments. These are then visualised as a network of clusters of nodes connected by lines (edges), with metabolites that share similar MS/MS fragmentations clustering together as a “molecular family” [26]. GNPS has its own internal MS/MS spectral libraries supplemented by data uploaded by GNPS users. During the process of molecular networking, the submitted MS/MS data are compared to these libraries with a view to fast-tracking the detection of known compounds. Additionally, within clustered molecular families, chemical relationships provide valuable structural information. For example, differences in molecular weights of 14 Da between two nodes could suggest CH_2_ homologues, whereas a difference of 16 Da could suggest an oxygenated analogue [26,27]. As outlined below, we have successfully utilised GNPS molecular networking to identify microbes that produce new analogues of rare structure classes (e.g., scopularides), as well as entirely new structure classes (e.g., amaurones).

**Figure 10:**
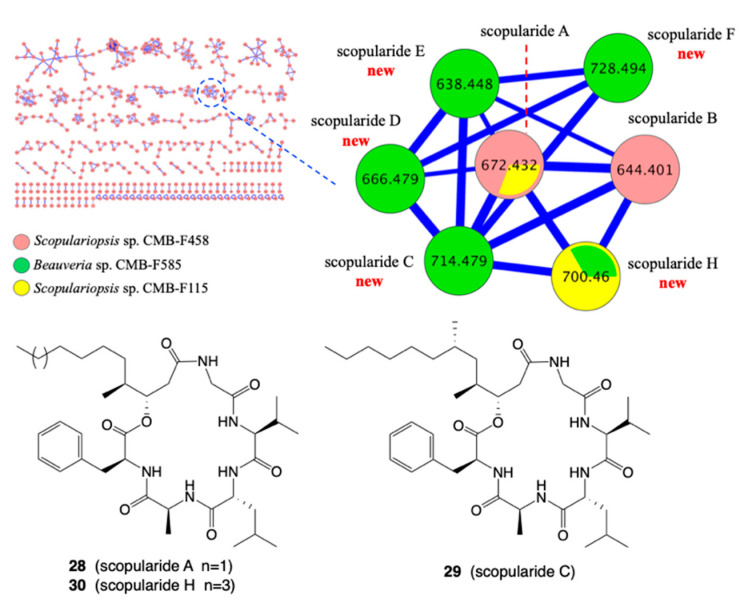
GNPS network for a marine fish-derived microbe library, with a unique cluster expanded for lipodepsipeptide scopularides and structures for scopularides A, C and H (**28**–**30**).

*A. Identification of new analogues of a known structure class:* During a GNPS molecular networking analysis of a marine fish-derived microbial library, we detected rare but known lipodepsipeptides including scopularide A (**28**) from *Scopulariopsis* sp. CMB-F458. A comparative GNPS molecular networking analysis of ×63 fungi co-isolated from the same fish gastrointestinal tract detected two additional fungi with co-clustering GNPS nodes attributed to new scopularides. During a follow-up study, *Beauveria* sp. CMB-F585 yielded scopularides C (**29**) to G, while *Scopulariopsis* sp. CMB-F115 yielded scopularide H (**30**) (Figure 10) [28].

**Figure 11:**
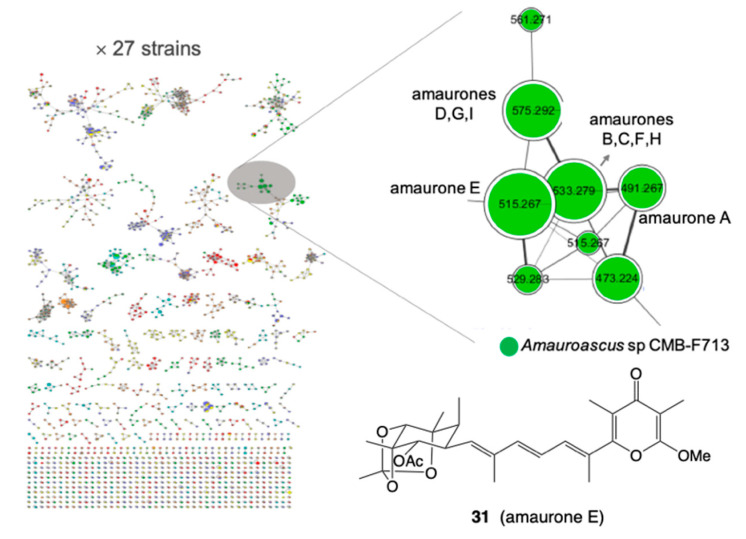
GNPS analysis of ×27 microbes, with expansion of a cluster unique to *Amauroascus* sp. CMB-F713 (green), and the structure of amaurone E (**31**).

*B. Identification of a new scaffold:* A GNPS analysis of a selection of ×27 microbial extracts revealed a cluster unique to the fungus *Amauroascus* sp. CMB-F713 (Figure 11) [29]. Large scale cultivation followed by purification and structural elucidation yielded a family of polyketide pyrones, amaurones A–I, featuring an unprecedented carbon skeleton, including the orthoester amaurone E (**31**).

## 6. Cultivation Profiling

### 6.1. Media Matrix

The media and conditions used to culture microbes in the laboratory are by necessity quite different to those encountered in the natural world. It is important to appreciate the importance of culture conditions as they play a key role in determining whether defensive natural products are produced or not. For example, in complex natural communities, a given species of microbe will undoubtedly encounter and respond to a multitude of chemical signals from adjacent microbes, with some triggering transcriptional activation of otherwise silent biosynthetic gene clusters (BGCs) encoding for ecologically important natural products that enhance survival (i.e., fight off adjacent competing species of microbe). Knowledge of these chemical signals can be pivotal to accessing the products of silent BGCs [30,31]. In addition to chemical signals, the transcriptional status of microbial natural products can also be influenced by other factors, including the diversity and concentration of media nutrients, oxygen levels, pH, light, metal ions, solid versus broth static versus broth shaken format, salinity, scale, cell density and culture maturity [32]. At a practical level, natural product profiles can be sensitive to the most innocuous of changes in media preparation, for example the difference between tap and distilled water [33]. Exploring the impact of microbial culture conditions can be an important strategy to accessing new natural products [34].

Notwithstanding that trialling many different culture conditions can yield new chemistry, when applied to large numbers of traditional solid and flask fermentations, this approach can be highly demanding of lab space, consumables and personnel. In an effort to ameliorate these costs, we have adopted a miniaturised 24-well microbioreactor approach known in the lab as the MATRIX. The MATRIX allows rapid reproducible cultivations under multiple media conditions, in shaken and static broths (1.5 mL) and solid phase (1.5 g). To microbioreactor broth cultivations, a serological pipette is used to add 1.5 mL of broth to each well, while, for solid-phase cultivations, 2 mL of warm agar is added to each well and the plate cooled to room temperature at an angle of 45° to form slants with larger surface area. Following incubation, in situ solvent extraction with EtOAc allows for rapid assembly of an extract library, which in turn facilitates high-throughput chemical and biological profiling [16,35].

**Figure 12:**
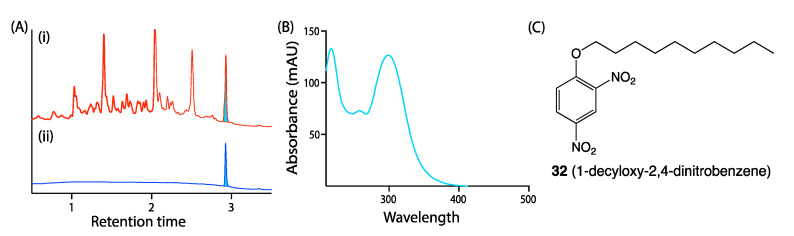
(**A**) UPLC–DAD chromatogram (1 μL injection) of (i) microbial extract (5 mg/mL) + **32** (50 μg/mL) and (ii) **32** (50 μg/mL) only (blue: calibrant peak); (**B**) UV spectrum of **32** and (**C**) structure of **32**.

**Figure 13:**
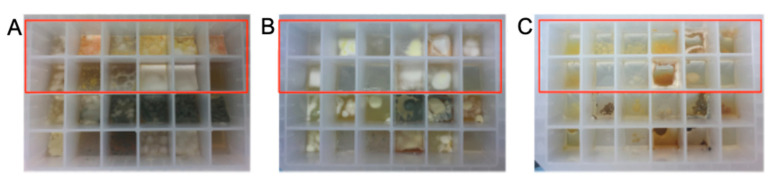
Photographs of 24-well microbioreactors: (**A**) solid agar, (**B**) broth static and (**C**) broth shaken inoculated with *Talaromyces* sp. CMB-TU011 cultivated in ×33 conditions (wells highlighted in red box).

**Figure 14:**
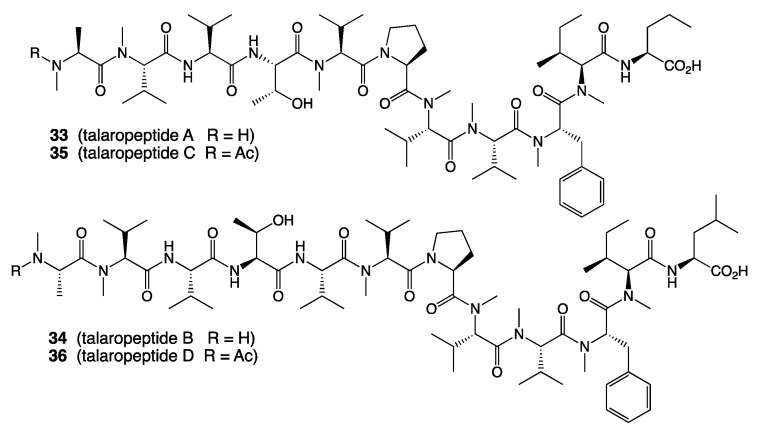
Structures of talaropeptides A–D (**33**–**36**).

**Figure 15:**
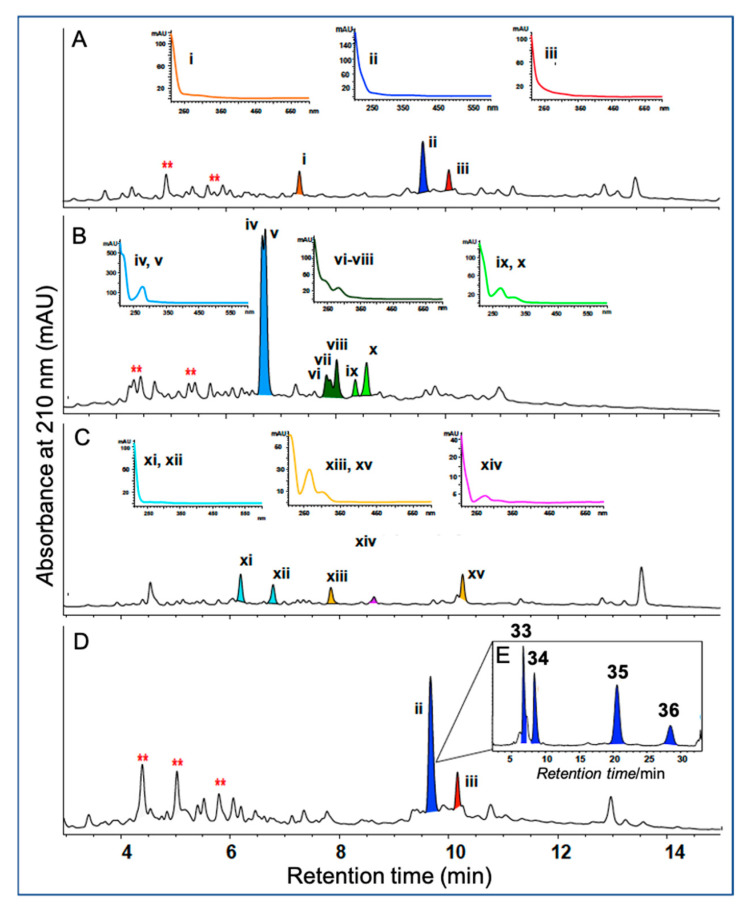
Representative HPLC–DAD (210 nm) chromatograms of *Talaromyces* sp. CMB-TU011 cultured on different media: (**A**) ISP-2 agar, (**B**) M2 shaken broth, (**C**) PYG agar and (**D**) YES static broth, with inset (**E**) showing talaropeptides A–D (**33**–**36**) resolved after HPLC method development (Zorbax SB-C3 column, with isocratic 40% MeCN/H_2_O inclusive of an isocratic 0.01% TFA/MeCN modifier). Roman numerals i-xv indicate detectable natural products. Metabolites with similar UV–vis profiles are coloured. ** Media background.

Chemical analysis of sets of MATRIX extracts helps identify and prioritize optimal scale-up culture conditions. For example, EtOAc extracts recovered from individual wells are concentrated under nitrogen to dryness then redissolved in a fixed volume of MeOH (1 mL) containing a known concentration of an internal synthetic calibrant. Our calibrant of choice, 1-decyloxy-2,4-dinitrobenzene (**32**), is readily synthesised, has a distinctive UV–vis chromophore and molecular weight, and a late retention time on reversed-phase chromatography that allows it to be readily detected but easily distinguished from natural products (Figure 12). The calibrant serves as an internal reference, facilitating quantitative comparisons of natural product production under different culture conditions. We also make direct comparisons to negative controls prepared from extracts of each uninoculated media (again with calibrant). A chemical analysis of MATRIX extracts can be performed via UPLC–DAD, or UPLC–QTOF–MS/MS with GNPS molecular networking. Our application of the MATRIX approach has been highly successful in revealing optimal scale-up culture and the targeting of a wealth of novel nature products. Some examples are shown below:

*A. MATRIX cultivation of Talaromyces* sp. CMB-TU011: An Australian marine tunicate-derived fungus, *Talaromyces* sp. was subjected to MATRIX cultivation, using a combination of ×11 different media and ×3 phases (i.e., solid agar, as well as static and shaken broth) (Figure 13), supported by UPLC–QTOF profiling, to reveal conditions for producing a new class of extensively *N*-methylated 11 or 12 residue linear peptides, talaropeptides A–D (**33**–**36**) (Figure 14) [36]. HPLC–DAD analysis of the MATRIX extracts clearly showed the productions of different metabolites under different conditions, with YES static broth condition being optimal for the production of talaropeptides (Figure 15).

**Figure 16:**
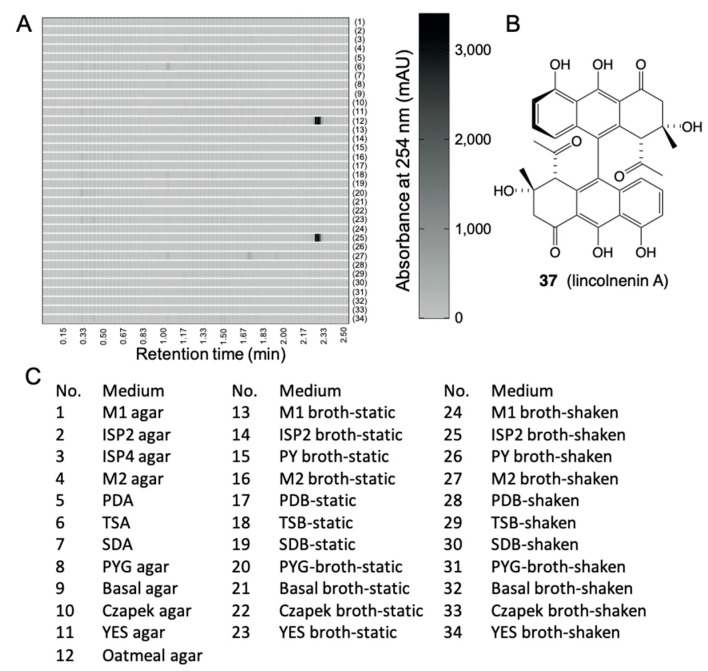
(**A**) Heat map of UPLC–DAD (254 nm) chromatograms of *S. lincolnensis* ACM-4234 cultured in ×34 different conditions (black cells represent lincolnenin production). (**B**) structure of lincolnenin A (**37**). (**C**) List of media conditions.

*B. MATRIX cultivation of Streptomyces lincolnensis:* ACM-4243: MATRIX cultivation analysis of *S. lincolnensis* on ×34 different culture conditions revealed that lincolnenins A (**37**)–D are only produced in two specific conditions: oatmeal agar and ISP2 broth shaken (Figure 16) [37].

**Figure 17:**
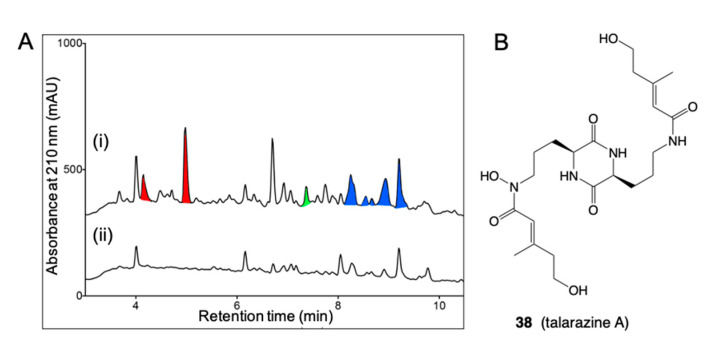
(**A**) HPLC–DAD (210 nm) chromatograms of *Talaromyces* sp. CMB-W045 in (i) jasmine rice media and (ii) red rice media. The siderophores talarazines (red peaks) were detected in jasmine but not red rice cultivations. (**B**) Structure of talarazine A (**38**).

*C. Red versus Jasmine Rice:* When grown on jasmine rice media known to be deficient in Fe(III), *Talaromyces* sp. CMB-W045 isolated from a mud dauber wasp collected from an urban location in Brisbane, Queensland, returned a new family of very potent siderophores, talarazines (i.e., talarazine A (**38**)). However, when grown on red rice known to be rich in Fe(III) *Talaromyces* sp. CMB-W045 failed to produce any trace of talarazines (Figure 17). This effect could be reversed by adding supplementary Fe(III) to jasmine rice cultivations (suppressing talarazine biosynthesis), or adding the Fe(III) chelator EDTA to red rice cultivations (activating talarazine biosynthesis). That the biosynthesis of the siderophore talarazines is linked to the availability of Fe(III) serves to illustrate that all rice media are not the same, and that Fe(III) is a transcriptional activator [38].

### 6.2. Large-Scale Cultivation

After a strain has been de-replicated and prioritised for chemical analysis, and a MATRIX approach has been used to identify optimal culture conditions, it is important to check the reproducibility of production of target natural products under scale-up conditions (i.e., moving from a 24-well plate analytical scale to an agar plate or a flask). Another factor to be studied before proceeding to full scale-up production is the optimum incubation time. This can be achieved by comparative HPLC analysis (with calibrant) of fixed aliquots from broth cultures, or agar plates, extracted at different incubation intervals (e.g., 8, 10, 12, 14 days, etc.), with the detected yield of target natural products determining both the duration needed for a successful scale-up cultivation, and the number of agar plates/flasks required. Even then, when proceeding to scaled-up production, we recommend the use of several “scout plates” or “scout flasks” two days ahead of the bulk cultivation. Scout plates can be harvested and analysed at key time points, to monitor natural product yields, and ensure that full-scale harvest occurs at an optimal time. Scale up cultures harvested too early risk lower yields, while cultures left too long risk catabolism of natural products with associated reductions in yield.

## 7. Microbial Natural Products

### 7.1. Extraction

The extraction of scaled-up solid-phase agar plate cultivations (i.e., 100+ plates) is typically achieved by harvesting the cellular biomass + agar into a single flask, adding organic solvent (i.e., ethyl acetate) into the chopped agar (1 × 1 cm^2^), followed by shaking overnight at 190 rpm before decanting the solvent and removal of solvent in vacuo at <40 °C. Broth cultures are subjected to liquid–liquid extraction by simply extracting the neat broth with equal volume of organic solvent (i.e., ethyl acetate) and performing a liquid–liquid partition in a separating funnel before concentrating the organic phase to dryness in vacuo at <40 °C. The resulting dry extracts are weighed and kept away from direct sunlight, sealed, labelled and stored in a −20 °C freezer prior to further fractionation.

### 7.2. Fractionation

**Figure 18:**
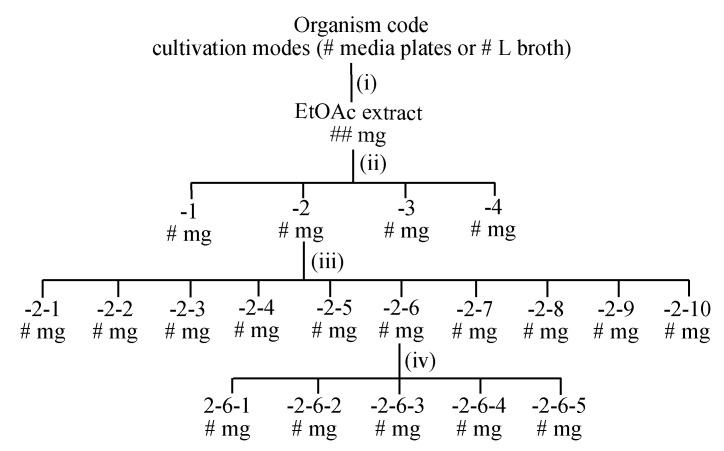
Best practice chemical fractionation involving (i) solvent extraction/partitioning; (ii) sequential solvent trituration to recover hexane (-1), CH_2_Cl_2_ (-2), MeOH (-3) and, where necessary, water (-4) solubles; (iii) preparative scale chromatography, using either solid-phase extraction cartridges or size exclusion “gel” chromatography (e.g., Sephadex LH20 or G10) or preparative reversed phase HPLC (e.g., C_18_ or C_8_); and (iv) if needed, semi-preparative or analytical scale HPLC. Note: all extracts, fractions and pure compounds are dried in vacuo or under dry nitrogen at <40 °C to facilitate mass, chemical and biological profiling.

There are a multitude of approaches that could be taken to fractionate a microbial extract. We prefer a minimalist approach that uses the least number of steps with the least stress on microbial chemistry. To achieve this, wherever possible we avoid acidic chromatography media, solvents and additives (i.e., silica, CHCl_3_ and TFA), as well as chemically reactive solvents (i.e., acetone); keep all fractions and compounds away from direct sunlight, sealed, labelled and in the dark in a −20 °C freezer when not in use; and to avoid thermal transformations, dry samples in vacuo or under dry nitrogen at <40 °C. Importantly, we record the mass of every fractionated sample to confirm mass recovery at each step, and record UPLC–DAD–MS chromatograms and ^1^H NMR spectra to confirm chemical integrity (i.e., to be alert for and not overlook unexpected transformations induced during handling, see Section 7.4, below). Where relevant, we also use quantitative bioassays to track bioactives across the fractionation process. Over many years, we have arrived at a laboratory best-practice fractionation scheme which allows us to routinely recover pure natural products in three or four steps (Figure 18).

This scheme can vary on occasion depending on (a) the mass of extracts and fractions (i.e., for small quantities we might exclude step iii), (b) the number of different natural product classes encountered in a crude extract (i.e., for extracts containing multiple structure classes with significantly different chemical and/or physical properties we may need 2 or 3 cycles of steps iii and iv), and (c) the chemical stability of natural products (i.e., if the mass, chemical or biological profiling reveals unexpected losses or transformations during steps iii and iv, these need to be revised and repeated).

### 7.3. Structure Elucidation

The remarkable powers of modern chemical and spectroscopic methods in structure elucidation have been the subject of many reviews and books; therefore, they are not elaborated upon here. Suffice to say, modern microbial biodiscovery has been the beneficiary of many technological advances in this area, such that it is now routine to solve full 3D chemical structures with as little as 1 mg of a new natural product. This is not to say that the application of this technology does not require the skill and ability of researchers fully conversant with the principles of organic chemistry, and with specialist knowledge and expertise in natural products chemistry, biosynthesis and spectroscopy. Quite the contrary. The effective use of modern methods in structure elucidation are heavily predicated on the skill of the practitioner, as evident in the many microbial natural product studies represented as examples in this review, and in the scientific literature.

### 7.4. Artifacts

**Figure 19:**
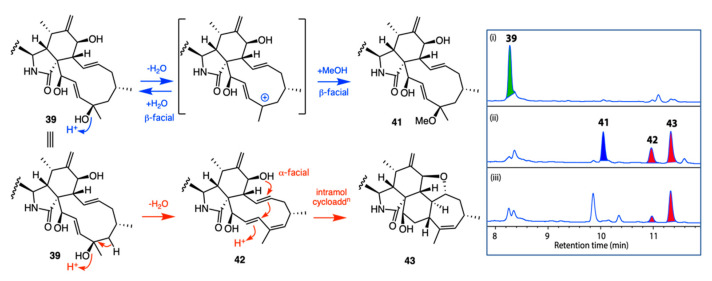
Proposed mechanism for acid-mediated conversion of **39** to **41**–**43**. Inset: HPLC–DAD (210 nm) chromatograms of cytochalasin J (**39**) (i) in MeOH, (ii) in 1% TFA in MeOH for 20 h and (iii) in 1% TFA in H_2_O for 20 h.

**Figure 20:**
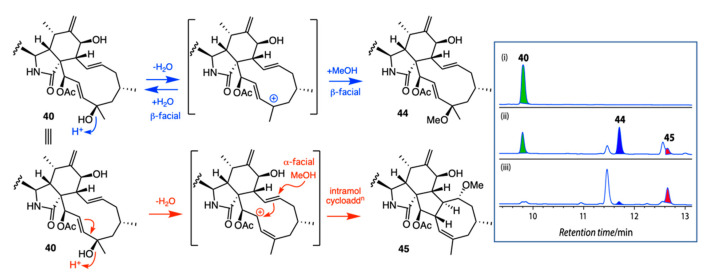
Proposed mechanism for acid-mediated conversion of **40** to **44** and **45**. Inset: HPLC–DAD (210 nm) chromatograms of cytochalasin H (**40**) (i) in MeOH, (ii) in 10% TFA in MeOH for 20 h and (iii) in 50% TFA in H_2_O for 20 h.

Based on many decades of experience, we have adopted the practice of securing a chemical analysis of fresh extract (i.e., UPLC–QTOF), and of retaining a portion of extract for comparative analysis with isolated pure chemicals to differentiate natural products from possible handling artifacts. We recommend re-analysing the LCMS chromatograms of the original extract, using a single-ion extraction (SIE) approach to detect (or not) the presence of isolated compounds. If the isolated compound cannot be detected in the extract, it is most likely an artifact. Another way to detect artifacts is to speculate on plausible biosynthetic origins for the natural products and the chemical mechanisms behind the transformation. A recent review documented natural product scaffolds most prone to generation of artifacts and provides mechanistic insights into their formation [39]. Once a potential artifact has been detected, analytical methods such as UPLC–DAD or UPLC–QTOF can be used to monitor the fate of any pure natural product exposed to conditions suspected to generate artifacts. Some noteworthy examples of artifacts reported from our lab are shown below:

*A. Acid Rearrangement of Cytochalasins: Phomopsis* sp. CMB-M0042F isolated from a marine sediment collected near Shorncliffe, Queensland, yielded the known cytochalasins J (**39**) and H (**40**) together with five new analogues, **41**–**45**. Failure to detect **41**–**45** in the fresh extract suggested they may be artifacts, possibly induced by the use of 0.01% trifluoracetic acid (TFA) during HPLC purification. This hypothesis was confirmed by treating **39** and **40** with TFA in MeOH (Figure 19 and Figure 20). The stereo and regiochemical control of the resulting acid-mediated intramolecular cycloaddition was remarkable, with the 21-OH moiety in **39** yielding the 5/6/6/5/7-fused ring scaffold evident in **43**, and the 21-OAc moiety in **40** yielding the 5/6/5/8-fused ring scaffold evident in **45** [40]. Knowledge of such functional group control over acid-mediated intramolecular cycloadditions has the potential to inspire novel biomimetic syntheses, enabling access to new cytochalasin scaffolds.

**Figure 21:**
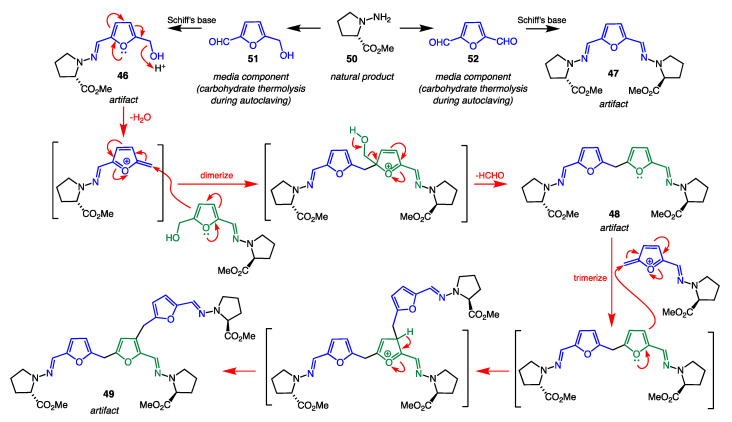
Acid-mediated mechanism for the formation of prolinimines A–D (**46**–**49**) from the fungus natural product **50** and rice media components **51** and **52**.

*B. Prolinimine Schiff Bases:* Prolinimines A–D (**46**–**49**) were first reported in 2018 from a fish gastrointestinal tract-derived fungus *Trichoderma* sp. CMB-F563 (later reclassified as *Evlachovaea* sp. CMB-F563). At that time, it was noted that while standard isolation and handling techniques applied to CMB-F563 rice grain media cultivations yielded prolinimines B–D (**47**–**49**), HPLC analysis of fresh EtOAc extracts prior to any fractionation detected only prolinimines A and B (**46** and **47**). These observations prompted speculation that **46**–**47** were natural products, but **48** and **49** were artifacts [41]. Subsequent investigations into the biosynthetic origins of the prolinimines revealed that, during solvent extraction of the rice media, *N*-amino-l-proline methyl ester (**50**) was released from the mycelia and rapidly reacted through Schiff’s base condensation with furans **51** and **52** (both are thermolysis artifacts of the rice media) to form the prolinimines (Figure 21) [42]. The latter finding demonstrates that, although prolinimines A and B (**46** and **47**) can be detected in fresh cultivation extracts, they are nevertheless artifacts of the solvent extraction process, with the sole natural product being *N*-amino-l-proline methyl ester (**50**).

**Figure 22:**
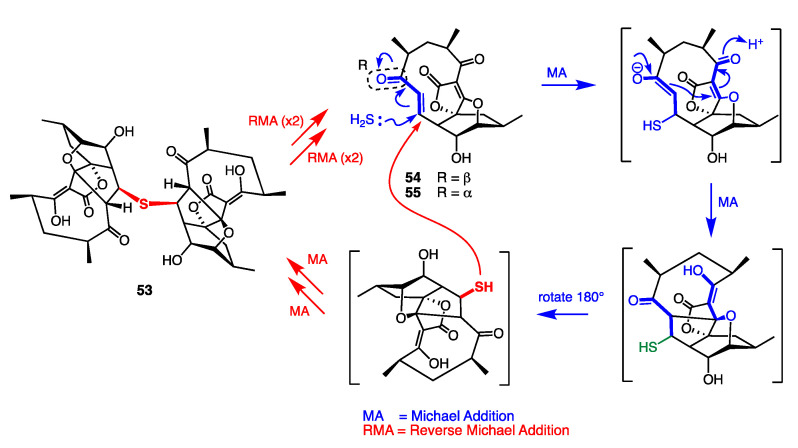
Quadruple reverse Michael addition of abyssomicin J (**53**) to *atrop*-abyssomicin C (**55**).

*C. Abyssomycin and Reverse Michael Additions:* Antitubercular bioassay guided fractionation *Verrucosispora* sp. MS100128 isolated from a South China Sea deep-sea sediment afforded the unprecedented natural product abyssomicin J (**53**), as a thioether adduct of abyssomicin C (**54**) [43]. Although prior studies had shown that abyssomicin C (**54**) and *atrop*-abyssomicin C (**55**) were anti-TB due to their Michael acceptor reactivity, we were intrigued to observe the anti-TB properties of abyssomicin J (**53**), even though it was not a Michael acceptor. This prompted the discovery of **53** as a prodrug, undergoing in situ (intracellular) oxidation of the thioether to a sulfone, followed by a quadruple reverse Michael addition to deliver two molecules of anti-TB *atrop*-abyssomicin C (**55**) (Figure 22).

**Figure 23:**
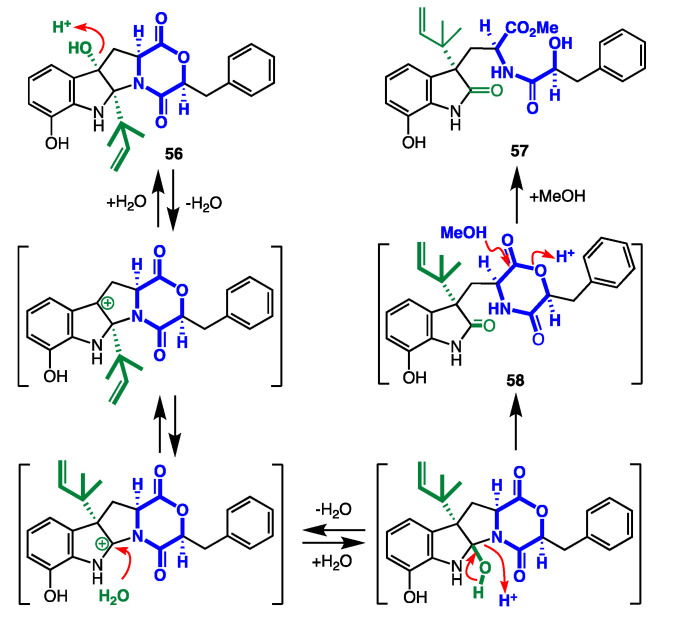
Proposed mechanism for the transformation of shornephine A (**56**) to *seco*-shornephine A methyl ester (**57**).

*D. Shornephine A Solvolysis: Aspergillus* sp. CMB-M081F isolated from a marine sediment near Shorncliffe, Queensland, Australia, yielded the diketomorpholine shornephine A (**56**). Of note, the natural product **56** was obtained following reversed-phase HPLC (MeCN/H_2_O) purification, while reversed-phase HPLC (MeOH/H_2_O) purification afforded the artifact *seco*-shornephine A methyl ester (**57**) [44]. The mechanism for the artifact formation can be explained by an auto (C-9 phenol) acid-catalysed dehydration to yield a C-4 carbocation, followed by a 1,2-sigmatropic rearrangement and H_2_O addition to yield *seco*-shornephine A (**58**), which was unstable and was transformed rapidly to the methyl ester **57** (Figure 23). Shornephine A is a non-cytotoxic inhibitor of P-glycoprotein (P-gp) mediated drug efflux in multidrug-resistant human colon cancer cells. Based on this discovery, synthetic diketomorpholines were prepared to probe P-gp structure-activity relationships and reactivity to solvolysis.

## 8. Future of Microbial Biodiscovery

Microbial biodiscovery is poised for a renaissance as new technologies and methodologies advance disciplines and reveal new insights into the hidden, and largely untapped chemical diversity encoded within the global microbiome. The challenge ahead is to continue to adapt, to define and to redefine best practice; to discover and document knowledge of the chemical and biological properties of microbial natural products; and to apply this to solve important problems in science, health, agriculture, the environment and society. With over a billion-year experience at refining and deploying chemicals to enhance survival, and having inspired some of the world most successful and profitable pharmaceuticals and agrochemicals, microbes have so much more to offer to those willing to take up the challenge.
